# Letter and Word Processing in Developmental Dyslexia: Evidence from a Two-Alternative Forced Choice Task

**DOI:** 10.3390/children12050572

**Published:** 2025-04-29

**Authors:** Daniela Traficante, Pierluigi Zoccolotti, Chiara Valeria Marinelli

**Affiliations:** 1 Department of Psychology, Università Cattolica del Sacro Cuore, 20123 Milano, Italy; 2 Scientific Institute, IRCCS E. Medea, 23842 Bosisio Parini, Italy; 3 Department of Psychology, Sapienza University of Rome, 00185 Roma, Italy; pierluigi.zoccolotti@crtspa.it; 4 Tuscany Rehabilitation Clinic, 52025 Montevarchi, Italy; 5 Cognitive and Affective Neuroscience Lab, Department of Humanities, Letters, Cultural Heritage and Educational Studies, Foggia University, Via Arpi, 155–176, 71121 Foggia, Italy; chiaravaleria.marinelli@unifg.it

**Keywords:** visuo-orthographic processes, developmental dyslexia, Reicher–Wheeler paradigm, letter processing, letter search, lexical activation

## Abstract

Background/Objectives: The present study aimed to investigate letter processing in children with dyslexia and typically developing readers as a function of the type of orthographic context. Methods and Results: In Experiment 1A, children performed a two-alternative forced choice task (Reicher–Wheeler paradigm) using as probes either high-frequency words, pronounceable pseudo-words, or unpronounceable non-words. The group differences in letter recognition were clearly distinguished from those present in typical word and pseudo-word reading conditions (Experiment 1B), as a global factor was present only in the latter case. In Experiment 2, the two-alternative forced choice task required the child to search for the target letter in the subsequent multi-letter string (i.e., words, pseudo-words, or non-words), thus reducing the memory load. Detecting the target letter was more difficult in a word than in a pseudo-word or non-word array, indicating that the word form’s lexical activation interfered with the target’s analysis in both groups of children. In Experiment 3, children performed the two-alternative forced choice task with symbols (Greek letters) either in the Reicher–Wheeler mode of presentation (Experiment 3A) or in the search condition (Experiment 3B). Children with dyslexia performed identically to typically developing readers in keeping with the selectivity of their orthographic difficulties. Conclusions: The present data indicate that children with dyslexia suffer from an early deficit in making perceptual operations that require the conjunction analysis of a set of letters. Still, this deficit is not due to an inability to scan the letter string. The deficit is confined to orthographic stimuli and does not extend to other types of visual targets.

## 1. Introduction

In orthographies with consistent grapheme-to-phoneme correspondences, such as Italian (the orthography object of the present study), children with dyslexia read slowly, although in a relatively accurate way [1,2,3,4]. Eye movement recordings while reading texts or lists of words reveal very small and numerous saccades indicative of their inability to process words holistically [5,6,7]. Consequently, they engage in a sequential, fractionated encoding expressed by a strong dependency on the length of the target word [2,8]. According to the Dual-Route Cascaded (DRC) model [9], they appear to rely more on using the sublexical route, i.e., the reading procedure based on the segmentation of the written letter string in graphemes (e.g., d-o-g) and the conversion of graphemes into phonemes (e.g., /dOg/). That procedure is the first applied by pupils in learning to read, corresponding to the ‘Alphabetic’ phase described by Uta Frith in her model [10]. The consistency of mappings between orthography and phonology in shallow orthographies can lead to high accuracy rates independent of access to lexical representations, but it is time costly. For this reason, typically developing (TD) readers, even in languages with consistent orthographies, soon acquire lexical representations and learn to read through the so-called ‘lexical route’ suggested by the DRC model. In this case, words can be recognized at sight, as the letter strings would activate representations stored in the orthographic input lexicon. The consolidation of such representations is typical of the ‘Orthographic’ phase described by Uta Frith in her model of learning to read. There is evidence that Italian children with dyslexia also use lexical representations, as their reading performance is influenced by lexical variables, such as word frequency and neighborhood size. Specific deficits may be detected with low-frequency words, indicating some limitation in the expansion and flexibility of use of their orthographic lexicon. Short, high-frequency words are likely to be recognized at sight even by Italian children with reading difficulties; however, a prevalent reliance on sublexical procedures for low-frequency words is observed (e.g., [11]).

To account for the reading slowness of children with dyslexia, we applied the Rate and Amount Model (RAM) [12] and the Difference Engine Model (DEM) [13], tuned to detect global components in individual differences in timed tasks, typically using reaction time (RT) data. The first model (RAM) assumes that “the time to complete a task is equal to the ratio of the amount of processing performed and the rate (e.g., cognitive speed)” ([12], p. 778). In this view, the analysis of the linear relationship between the response latencies of children with dyslexia and TD children in different tasks can provide an estimate of the differences in the amount of information processing performed in a task, given their differences in overall processing speed. The second model (DEM) aims to face “the problem of how individual ability and task difficulty interact with other individual difference variables such as age and health status” ([13], p. 267). To solve this problem, the authors propose considering the linear relation between variability (standard deviation—SD) and mean reaction time (RT), as SDs are greater when RTs increase. The RAM and DEM models offer complementary information on the characteristics of a global factor: the first provides an estimate of the cognitive speed concerning the amount of processing required by a set of tasks; the latter highlights the presence of residual specific effects, over and beyond the presence of a global factor, i.e., the effect of the presence of dyslexia on the performance. A single global factor accounts for a large proportion of the group differences in all tasks concerning word and pseudo-word processing [14,15]: Italian primary-school children with dyslexia were impaired in dealing with letter strings compared to typically developing children progressively more as a function of difficulty of the condition but independently of the lexicality [14], frequency, and pronounceability of the target stimuli [15]. In keeping with the generality of the deficit, the global factor acted in both lexical decision and reading tasks, although with different slope parameters [14]. However, it was selective for orthographic materials, i.e., it was not present when children had to name pictures or when stimuli were presented in the auditory modality (e.g., [16]). While children with dyslexia show a severe deficit in all tasks involving the processing of visually presented multi-letter strings, they have a spared ability to process single letters, an observation early made by Katz and Wicklund [17,18]. Indeed, most evidence indicates that they are not impaired in processing letters if presented in an isolated fashion (e.g., [19]), even if the test is adaptively carried out, controlling for task difficulty. Consistently, unlike the case of words and pseudo-words, tasks requiring the naming or matching of individual letters, bigrams, or two-letter syllables do not contribute to the global factor [15]. Moreover, children with dyslexia showed reduced sensitivity also on a symbol-string task used to assess sensitivity to the position of briefly presented non-alphabetic but letter-like symbols [20]. Overall, quoted studies identifying a selective deficit in children with dyslexia share the task requirement that the entire string of orthographic (and possibly also of non-orthographic) stimuli be simultaneously processed to carry out the naming (or matching) response. By contrast, children with dyslexia are unimpaired in processing single letters or bigrams [15,18,19] or when the set of target letters is presented sequentially [21]. Based on these data, we tentatively proposed that the global factor marks the ability to represent the graphemic string (e.g., [16]). According to Marsh and Hillis [22], establishing a “graphemic description” represents an obligatory step in proceeding to the lexical/sublexical analysis of the word.

Some attempts have been made to understand the reason for the observed deficit in processing multi-letter strings. Bosse et al. [19] proposed that some children with dyslexia show a deficit in visual span (i.e., a limitation in the number of discrete visual elements, which can be processed in parallel in a visual display, independent of phonological skills) and that this visual span deficit marks a bottom-up sensory limitation on reading [23,24,25,26]. In fact, Lassus-Sangosse et al. [21] found the visual span deficit with the simultaneous presentation of letters but not when the same letters were presented in rapid sequence, indicating preserved serial processing skills in children with dyslexia.

According to Deheane et al. [27], orthographic strings are processed through a hierarchic activation of increasingly broader and more abstract local combination detectors (LCDs). At the last level of processing, “multiple letter detectors have to be replicated at several locations, thus forming the bank of case-invariant letter detectors postulated in many models” ([27], p. 337). In this view, a deficit in the activation of multiple letter detectors seems a likely candidate for the slowness deficit shown by children with dyslexia and/or for difficulty in acquiring the orthographic representations of words [28].

The picture is more complex if one considers the studies on processing an isolated target stimulus presented before or after a multi-element display. Thus, several investigations evaluated the performance of children with dyslexia in searching for a letter within a letter string or in identifying whether a target letter was present in a previously presented array of letters. Often, these tasks take the form of a two-alternative forced choice (2AFC) task requiring the child to identify the location of the target in relationship to the position of a probe (e.g., upper-lower, or response-limited mode [29]) or to identify the identity of the target in relationship to two alternatives (or data-limited mode; [30]). Both array-letter and letter-array sequences have been used [29,31].

However, to date, the picture emerging from these studies still delineates a complex pattern of findings. When matching a target to a visual array, children with dyslexia seem more affected by large than small arrays [6,32]. So, array size may have a role even in cases where no full report is required. However, this effect does not seem consistent with any visual or visual–spatial model of dyslexia [33,34]. When the influence of the serial position of the target on the letter array was studied systematically, no group differences were detected [34,35]. Finally, evidence seems inconclusive on the relative role of visual and phonological factors. Some studies found letter recognition to be influenced by visual similarity [31,32]. Others indicated that the dyslexic deficit was present with nameable stimuli (such as letters or digits) but not unfamiliar symbols [34,35], suggesting that impaired symbol–sound mapping rather than impaired visual–attentional processing is likely to be involved in poor reading performance.

The type of response itself may dramatically alter the pattern of results. At variance with two previous reports [6,30], Hawelka and Wimmer [33] found that differences between dyslexic and control readers disappeared when the verbal report was avoided in favor of a purely visual response. According to Ziegler et al. [34], the absence of group differences with symbols indicates that children are impaired whenever stimuli map onto phonological codes. Alternatively, it has been proposed that the lack of deficit for symbols can be interpreted based on stimulus familiarity [36]. In this line of thought, people are not used to reading strings of symbols, while letter and digit strings are familiar stimuli, and this feature makes them easier to process than symbols. To support this proposal, Valdois et al. [36] refer to their finding (from a study using the full-report paradigm) that children with dyslexia are unimpaired in naming strings of colors even though they are easily nameable stimuli.

As reading is related to the acquisition of an orthographic lexicon, one may want to examine whether the presence of a lexically laden context may influence letter recognition in children with and without dyslexia. The 2AFC procedure can also examine the interplay between lexical activation and graphemic processing, as in the Reicher–Wheeler paradigm [37,38]. In such a paradigm, a target letter is displayed after a word or pseudo-word. Results showed that the letter is easier to detect if preceded by a word than a pseudo-word (the so-called “Word Superiority Effect”). Furthermore, a “Pseudo-word Superiority Effect” (PSE) has also been frequently reported when a target letter is better recognized if it is preceded by a pronounceable pseudo-word than by an unpronounceable letter string (non-word) [39]. According to Grainger et al. [40], the PSE can be interpreted by referring to different aspects: (a) the role of lexical representations (lexical interpretation) as a consequence of a word misperception [41] or as word-letter feedback [42], (b) the pronounceability of pseudo-words (sublexical–phonological interpretation), or (c) to the familiarity of letter combinations in pseudo-words compared to non-words (sublexical–orthographic interpretation) [43].

Grainger et al. [40] used the Reicher–Wheeler paradigm with French children with and without dyslexia. They found a much larger PSE than WSE, with limited differences between the two groups of children, and concluded that, in processing words and pseudo-words, the sublexical–phonology interpretation was not a good explanation, as children with a phonological deficit also showed the same PSE effect as typically developing children. The authors proposed that the sublexical–orthographic hypothesis better accounted for their results. Following Massaro and Cohen ([43]; p. 439): “According to this account, letter perceptibility is improved when the adjacent letters form a typical orthographic context for a given letter in a given position”. This explanation is consistent with the studies that support the role of the detection of visual–orthographic patterns in the usual processing of known and new words and show that the Reicher–Wheeler paradigm can be a helpful tool to shed light on the mechanisms underlying letter string processing [44].

It is worth noting that the role of visual–orthographic components and phonological mapping between graphemes and phonemes on letter-string processing might be modulated by the consistency of the orthography individuals are familiar with. Marinelli et al. [15] tested the WSE and PSE in a sample of Italian dyslexic children and typically developing readers, finding that both groups showed the PSE. This result is consistent with the assumption that in young Italian readers—irrespective of their reading skills—accuracy in letter discrimination is influenced by the ortho-phono-tactic regularity of the letter string. Moreover, the advantage in letter recognition on a word compared to a pseudo-word context (WSE) was significant only for children with dyslexia. This result is consistent with several Italian studies (e.g., [11]), which showed lexical involvement in reading by children with and without dyslexia. Children with dyslexia were facilitated in performing the Reicher–Wheeler paradigm with stimuli that allow a higher lexical activation because lexical processing might support the sublexical one. On the contrary, for skilled readers of a shallow-orthography language like Italian, the sublexical route is likely to be a procedure effective enough to support reading of short stimuli like those used in the Reicher-Wheeler paradigm, irrespective to the backward activation from lexical entries, due to the fast application of simple and very consistent rules of conversion from graphemes to phonemes [45].

More recently, Mehlhase et al. [46], using a variant of the Reicher–Wheeler paradigm with four types of stimuli (words, legal pseudo-words, illegal pseudo-words, and non-word), did not find evidence for an automatized access to the orthographic lexicon in German 10-year-old children with and without reading and spelling deficits, as WSE was not detected in their group of participants. Only typically developing children (TD) showed higher accuracy with legal pseudo-words than illegal pseudo-words, showing their sensitivity to the typical orthographic context (according to the sublexical–orthographic hypothesis). Both TD and children with isolated reading fluency deficits showed their sensitivity to pronounceability of the string of letters (their N400 was higher for illegal pseudo-words than for non-words), whereas, in children with isolated spelling deficits and children with both reading and spelling deficits, this effect did not reach significance level, thus suggesting a reduced sensitivity for phonological word processing in these children.

## 2. The Present Study

Based on the reviewed evidence, the general aim of the present set of experiments was to investigate letter processing in interaction with different types of orthographic stimuli in children with dyslexia and in age-matched typically developing readers. We focused on the role of lexical activation, as well as on ortho-tactic regularity and pronounceability. Reading skills may be facilitated when detecting familiar patterns in letter strings (orthotactics), and children with dyslexia can take advantage of the distributional properties of letter combinations in the language, as typically developing children (e.g., [47]). Furthermore, by using symbols, we also examined the selectivity of the processing deficit for the presence of orthographic materials.

In Experiment 1A, we examined the WSE and PSE in Italian children with dyslexia and compared them to age-matched typically developing children. Testing the WSE effect would allow us to evaluate whether children with dyslexia can profit from the lexical pre-activation in successive letter recognition. Given the limited expansion of their orthographic lexicon, we used only high-frequency words. Based on the sublexical–orthographic hypothesis [40], testing for the PSE would allow us to evaluate whether children with dyslexia can profit from a pronounceable letter string, which forms a typical orthographic context in Italian. In a previous study [15], dyslexic children (and typically developing readers attending the same classes) performed a Reicher–Wheeler paradigm with mixed lists of words, pseudo-words, and non-words. In the present study (Experiment 1A), we used a Reicher–Wheeler paradigm experiment with separate blocks for the different types of contexts (i.e., words, pseudo-words, or non-words). Performing the task with non-lexical stimuli intermixed with words might induce a greater lexical activation for the former stimuli, producing a smaller WSE. Using a blocked list paradigm, we expected a weaker lexical activation for non-lexical stimuli than in Marinelli et al. [15] and possibly a significant WSE (Hypothesis 1).

Even though most studies on the Reicher–Wheeler paradigm focus on accuracy, parallel effects have also been reported with time measures (RTs and visual evoked potentials [46,48,49,50]). We were interested in examining the processing speed of children with dyslexia in responding to these tasks. We have previously shown that children with dyslexia are slow in reading words and pseudo-words and that their vocal reaction times (vRTs) grow as a function of stimulus difficulty over and above the influence of various experimental manipulations (e.g., [14]). However, this relationship was very weak in the case of letter, bigram, or two-letter syllable tasks. So, we wanted to test whether global components would intervene when letter recognition occurs in the context of a multi-element array. Data from Marinelli et al. [15] indicated that RTs in the Reicher–Wheeler paradigm showed no over-additivity effects, i.e., no increase in variability as a function of condition difficulty, as expected if global components were at work. Here, we wanted to test whether the same results would be present using a Reicher–Wheeler paradigm with blocked lists of words, pseudo-words, and non-words. To test this prediction, we examined whether group data based on the Reicher–Wheeler paradigm would fit the predictions of the DEM model [13], i.e., if a global factor was detectable in the data for the letter recognition task. For comparison, we also administered a task of reading words and pseudo-words (Experiment 1B) for which, based on previous evidence (e.g., [14]), we expected to find a global deficit in performance between the two groups of children (Hypothesis 2).

In Experiment 2, we devised a 2AFC search task: children had to detect the presence of a target letter in a subsequent string of letters. Thus, in this case, the task required detecting the target letter by searching it within the orthographic string, which again varied for lexical activation and pronounceability (i.e., words, pseudo-words, and non-words). The aim was to assess whether children with dyslexia have a deficit in scanning the orthographic string and if the type of orthographic array influenced the letter search in orthographic strings to the same extent as in typically developing children (Hypothesis 3). Note that this procedure has the advantage of minimizing the working memory load (only one letter to retain in memory instead of the four-letter stimuli) and testing the modulation of the context online (the string is present when children make their decision).

Finally, in Experiment 3, we evaluated whether children with dyslexia have a selective difficulty with orthographic materials and whether their difficulty also extends to non-orthographic stimuli (Hypothesis 4). The performance with symbols was examined with the classical Reicher–Wheeler mode of presentation (Experiment 3A; symbol recognition task) as well as with the 2AFC search task (Experiment 3B; symbol search task).

In all these experiments, we examined the effect of position. If children are applying a serial scanning of the stimulus, a strong letter position effect is expected, with an increasing percentage of errors from left to right. On the contrary, if they are trying to grasp the whole string, the position effect should be either nil or consistent with the W-shaped curve originally reported by Mason [29] and confirmed in children by Ziegler et al. [34] (Hypothesis 5).

## 3. Experiment 1A

Children were given the classical 2AFC Reicher–Wheeler paradigm, with word, pseudo-word, and non-word contexts to check the presence of the WSE and PSE in children with dyslexia and typically developing readers. Words, pseudo-words, and non-words were presented in three blocked lists, one for each type of stimulus.

### 3.1. Materials and Methods

#### 3.1.1. Participants

A total of 41 4th-grade children participated in the study: 17 children with dyslexia (10 M and 7 F; mean age: 9.50 years, SD = 0.30) and 24 typically developing readers (9 M; 15 F; mean age: 9.50 years, SD = 0.30). All children had nonverbal intelligence within normative values (according to the Raven Colored Progressive Matrices; [51]), and they lived in an adequate socio-educational context. Children were selected from local public schools during a screening for learning disabilities carried out by our laboratory. Typically developing children attended the same classes as dyslexic participants. This will ensure control for differences in participants’ educational opportunities. Parents authorized the participation of their children in the study. Trained examiners administered the assessment tools and scored the results.

Children with dyslexia were selected if they scored at least 2 SDs below normative values on accuracy and/or speed in a standard reading test (MT reading test; [52]). None of the children had received treatment for their reading impairment. All children with dyslexia were severely impaired in accuracy (mean z score = −2.99, SD = 1.19), and 59% were impaired in speed (mean z score = −1.75, SD = 1.25). Only 23% showed a performance in comprehension below normative data (mean z score = −0.27, SD = 0.43). Data on reading and cognitive performance are reported in Table 1. Typically developing children were selected for reading speed and accuracy within normative values on the MT reading test [52]: their mean z scores were near zero in both accuracy (z = −0.03, SD = 0.80), speed (z = −0.10, SD = 0.57), and comprehension (z = 0.04; SD = 0.26). The two groups were significantly different in reading accuracy (t (40) = 9.56, *p* < 0.0001), speed (t (40) = 5.73, *p* < 0.0001), and comprehension (t (40) = 2.84, *p* < 0.01). Groups were comparable for sex (Yates χ^2^ = 1.06, n.s.), age (t (40) = 0.08, n.s.), and Raven’s test performance (t (40) = 0.04, n.s.).

Other tests were used to qualify the reading and cognitive profile of children with dyslexia. Table 1 also reports the performance of the two groups of children on phonological and visual attentional span tests (for information on these tests, please see below). As a group, children with dyslexia showed lower performance than control readers in several of these tests, i.e., the Visual Attentional Span, the Repetition of Non-words Series, and at the Blending test in the pseudo-word condition (only a trend was present for the word condition).

The Visual Attention Span [19] is a task in which children see on the PC screen for 200 ms an unpronounceable string of five consonants (e.g., RHSDM) that, as such, cannot be coded phonologically and must report as many letters as possible. Each letter is presented 10 times, appearing twice in each of the five positions. The task includes twenty items and was implemented by the E-Prime 2.0 software.

In the Repetition of Non-word Series [53], ten lists of three bi-syllabic, five-letter pseudo-words are read aloud by the examiner at a pace of about one every 2 s. The child must repeat each list as accurately as possible immediately after the presentation. Each correct non-word was awarded a point for a maximum score of 30.

The Blending Test [53] measures phonological awareness. Words (or pseudo-words) are presented phoneme-by-phoneme through an audiotape at a rate of one per second. At the end of the sequence, the child must repeat the whole stimulus aloud. Nineteen (five-to-six-letter) words/pseudo-words are presented. For each stimulus, the correctly blended pairs of phonemes are counted, irrespective of whether the entire target is produced. The maximum score is 83. No difference was present in the Phonological span, which was assessed with the Digit span task of the WISC-III [54].

At any rate, it may be noted that, in most cases, the impairment was mild and (except for the pseudo-word condition of the Blending test) very few children showed frankly impaired performance in these tests. Overall, children with dyslexia, as a group, were mildly impaired in both visual string processing and phonological and meta-phonological tasks. There was no difference in short-term memory, as measured by the Digit Span task.

#### 3.1.2. Stimuli

We presented three groups of 4-letter stimuli: 48 words (e.g., VISO, face), 48 pseudo-words (e.g., VESI), and 48 letter strings (e.g., VRSN). Each pseudo-word or non-word was derived by maintaining two letters from the original word. All the selected words had a CVCV structure and were chosen from the Elementary lexicon by Marconi et al. [55] and were high-frequency words (M = 181/1 million, SD = 261) with a high degree of familiarity [56] (M = 6.8/7 rating scale points, SD = 0.08), and they were easily recognizable as legitimate Italian words (word likeness) [56] (M = 99.35% of correct lexical judgement by adult proficient readers, SD = 0.7), and with a mean of 4 orthographic neighbors [56] (M = 3.9, SD = 1.7).

We created pseudo-words by altering the two vowels of a base stimulus. The resulting letter strings were composed of legal bigrams, which are sequences of two letters found in genuine Italian words. We avoided specific bigrams like SC, GL, GN, and CH, as these combinations correspond to a single sound in the first and third positions. Target letters were selected as minimal phonological pairs—pairs of phonemes that differ by only one phonological feature (e.g., P–B, L–R, N–M)—to highlight the importance of phonological decoding. The competitor letter was never included in the multi-letter string, and, in the case of substitution in the string, the competitor did not produce a lexical orthographic neighbor of the target itself.

In each position, we matched the number of visually similar competitors (53%) (e.g., P–B, N–M). The target letters in the 2nd and 4th positions were always vowels. Thus, in this case, we could not use minimal pairs. Additionally, due to the ortho-phonotactic structure of the Italian language, where words typically end with a vowel (a, e, i, or o), stimuli with targets in the fourth position were presented but categorized as fillers. This was because, in the context of real words, they often form actual words, which was not the case for consonant targets. However, these stimuli were included to prevent children from concentrating solely on the first three letter positions.

For each type of stimulus, there were 16 targets in the first position, 16 in the second position, and 16 in the third position, totaling 48 stimuli per group and 144 stimuli overall. Additionally, there were 8 filler stimuli in each group, with the target in the fourth position, totaling 24 filler stimuli. This brings the overall number of stimuli to 168. Three blocks of stimuli were created, one for each type, with a brief pause in between to prevent attentional decline. The order of stimuli was randomized within each block, and the sequence of the blocks was randomized for each participant.

#### 3.1.3. Procedure

Children completed the task in a quiet room, seated approximately 54 cm from the screen. The stimuli were presented in Courier New font, size 18 pt, in upper case (on average 0.40° of visual angle) with a white foreground on a grey background. Such conditions were chosen following Ziegler et al.’s work [34], one of the key references of the present research.

**Figure 1:**
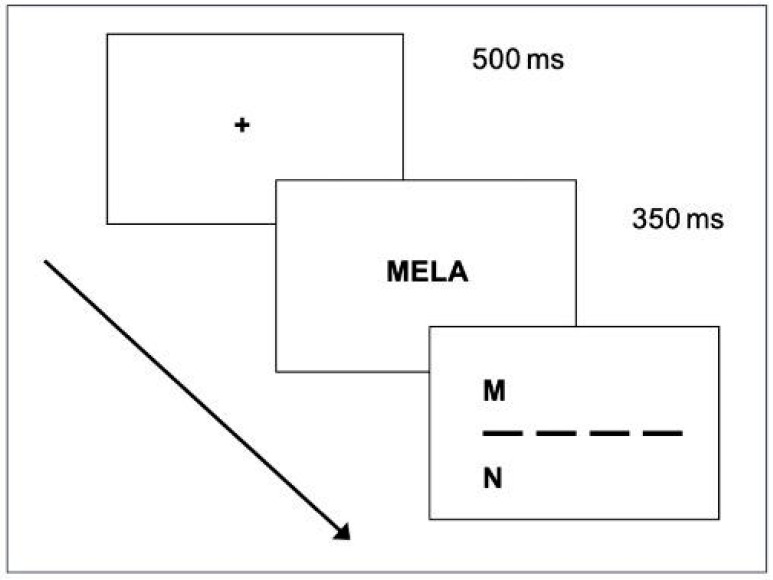
Experiment 1: Time-course of the trial.

The trial sequence began with a “get-ready” display lasting 500 ms, followed by a multi-letter string (either a word, pseudo-word, or non-word) for 350 ms. After this, the target letter display was shown, remaining visible until participants made a forced choice between the target and a competitor (as illustrated in Figure 1). The participant responded by pressing one of two buttons on the keyboard: the ‘Up’ button to choose the letter displayed in the upper part of the display and the ‘Down’ button for the letter in the lower part. In half of the cases, the correct response was the ‘Up’ choice.

At the beginning of the experimental session, ten training stimuli were presented. This was followed by three blocks of stimuli, presented in a fully randomized order, both between and within each block.

The program automatically recorded the participant’s responses. The dependent measures used were the percentages of errors and response times (RTs) for correct responses only. Outliers, defined as RTs that fell more than three standard deviations above the mean, as well as invalid responses—those with times faster than 250 ms or RTs that were not recorded accurately due to technical issues—were excluded from the analysis. In order to test the significance of the difference between the two groups of children, a measure of the effect size (ES) was carried out. Following Hedges’ [57] remark on the role of the sample size on effect size measures, Cohen’s *d* values were converted to Hedges’ *g* [58], an index also called Standardized Mean Difference. According to Cohen [59], the effect size (ES) can be considered small, if ES ≤ 0.3; moderate, if 0.3 < ES ≤ 0.49; and large, if ES ≥ 0.5.

### 3.2. Results

Invalid trials and outliers were 2.49% for children with dyslexia and 1.27% for typically developing children.

#### 3.2.1. Accuracy

The ANOVA on errors showed a significant effect of group (F_1,39_ = 3.82, *p* < 0.05, η^2^ = 0.09; SMD = 0.61): children with dyslexia made more errors (17.1%) than control children (11.4%). The main effect of context was significant (F_2,78_ = 51.05, *p* < 0.001, η^2^ = 0.57). A reliable PSE was found with more letter recognition errors in the non-word (23.4%) than in the pseudo-word (11.0%; *p* < 0.0001, Duncan test; SMD = 1.09) context. The WSE, i.e., the gain of lexical stimuli on pseudo-words, fell short of significance (word context: 8.32%; *p* = 0.09). The group did not interact with context (F_2,78_ < 1, n.s.): the WSE was negligible in both dyslexic and typical readers, and the PSE was significant and similar in both groups (*p* < 0.0001; typical readers: SDM = −0.99; children with dyslexia: SDM = −1.29).

The main effect of position (F_2,78_ = 41.29, *p* < 0.001, η^2^ = 0.51) was significant: letter recognition errors increased from the first (9.5%) to the second (13.8%) and third (19.5%) position (the fourth position was used only as a filler). Position influenced performance in interaction with the group factor (F_2,78_ = 7.54, *p* < 0.001, η^2^ = 0.16), with a significant group difference only in the third (*p* < 0.01; SMD = 1.05), but not in the first or second position (n.s.). Typically developing children had similar percentage of errors in the second and third positions, with only the latter being higher than the first position (*p* < 0.001; SMD = 0.66); children with dyslexia showed progressively low accuracy, passing from the first to second position (*p* < 0.05; SMD = 0.37) and from the second to third position (*p* < 0.0001; SMD = 1.02).

**Figure 2:**
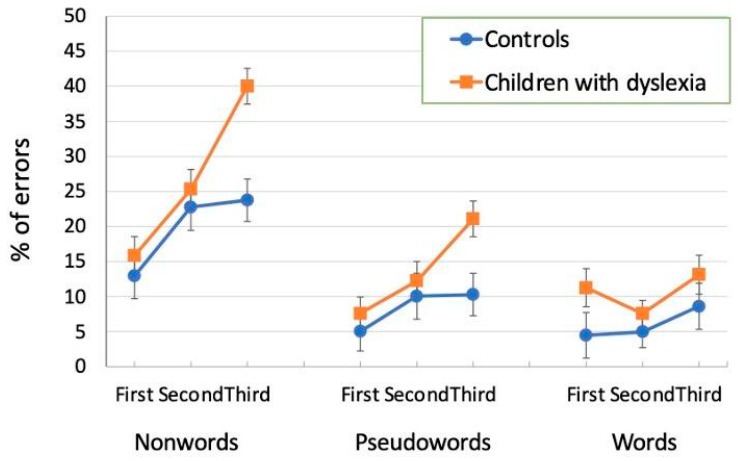
Group by context by position interaction on percentages of errors (Experiment 1A).

The context by position interaction was significant (F_4,156_ = 10.01, *p* < 0.001 η^2^ = 0.20) as well as the three-way group by context by position interaction (F_4,156_ = 2.93, *p* < 0.05, η^2^ = 0.07) was significant. The relevant means of this latter interaction are presented in Figure 2. An inspection of the figure indicates that the effect of position on letter recognition was similar in the two groups of children in the word context but not in the non-word and pseudo-word contexts. For children with dyslexia, there was a strong serial position effect in both the non-word and pseudo-word contexts: in the former, there was an increasing number of errors from the first to second position (first: 15.8%; second: 25.3%; *p* < 0.001; SMD = 0.71), and from the second to third position (40.0%; *p* < 0.001; SMD = 1.09). The pseudo-word context showed a similar trend, although with generally fewer errors: the difference from the first (7.6%) to the second position (12.2%) was not reliable but only from the second to third (21.1%, *p* < 0.001; SMD = 0.68) position. In the word context, there was no difference between the first and second positions (first: 11.3%; second: 7.6%, n.s.), but only between the second and third positions (13.1%, *p* = 0.05; SMD = 0.43). For control children, the effect of position was present in the non-word context and limited to the difference between the first (12.9%) and the second (22.8%) and third positions (23.7%; both *p* < 0.001; SMD_1vs2_ = 0.7; SMD_1vs3_ = 0.8); the last two did not differ from each other. For both the pseudo-word and word contexts, no reliable significant difference among positions emerged in these children.

#### 3.2.2. Reaction Times

The ANOVA on RTs showed no main effect of group (F_1,39_ = 1.26, n.s., η^2^ = 0.03) and context (F_2,78_ = 0.05, n.s., η^2^ = 0.00). The main effect of position (F_2,79_ = 27.97, *p* < 0.0001, η^2^ = 0.49) indicated longer RTs passing from the first (1076 ms) to the second (1304 ms, *p* < 0.0001; SMD = 0.61) position and from the second to the third (1400 ms) position (*p* < 0.01; SMD = 0.23). The context-by-position interaction was significant (F_4,156_ = 2.82, *p* < 0.05, η^2^ = 0.06). RTs were slower for the second than the first position for all three types of contexts (non-words: SMD = 0.61; pseudowords: SMD = 0.47; words: SMD = 0.48). Slower RTs for the third than the second position were present for the non-word context (second position: 1322 ms; third position: 1440 ms, *p* < 0.001; SMD = 0.22) and the pseudo-word context (second position: 1271 ms; third position: 1452 ms, *p* < 0.001; SMD = 0.32) but not the word context (second position: 1288 ms; third position: 1323 ms, n.s.). All other interactions were not significant.

## 4. Experiment 1B

In this experiment, children performed a word and pseudo-word reading task. Previous research has shown that this type of task can help identify global components in the data that account for a large proportion of the differences between children with dyslexia and typically developing readers [12,14]. Our first aim was to confirm this basic finding. Secondly, we investigated the presence of global components in the data using the Reicher–Wheeler paradigm and whether these components could be aligned with the group differences expected in the reading task. By examining both datasets together, we sought to determine whether they could be attributed to the same global factor.

### 4.1. Materials and Methods

#### 4.1.1. Participants

Same as Experiment 1A.

#### 4.1.2. Stimuli

Stimuli were selected from Marinelli et al. [60,61] to evaluate the effects of length (4 and 6 letters) and stimulus type (high-frequency words, low-frequency words, and pseudo-words), resulting in a total of 10 stimuli in each subset. Only words with regular stress patterns, specifically those with stress on the penultimate syllable, were included in the word set. High-frequency words had a mean frequency of 135.0 (SD = 89.6) over 1 million occurrences, while low-frequency words had a mean frequency of 3.6 (SD = 4.6). These values were derived from the child frequency count reported by Marconi et al. [55]. The sets were balanced for various factors, including bigram frequency, N-size, ortho-syllabic difficulty (the presence of double consonants, clusters of consonants), contextual rules (see [62]), phonetic features of the initial phoneme [63], and word frequency. Pseudo-words were generated by altering one to three letters of high-frequency words, ensuring they maintained the same ortho-syllabic difficulty as legal words, including the presence of double consonants, consonant clusters, contextual rules, and bigram frequency.

#### 4.1.3. Procedure

The child had to read the stimulus on the screen as quickly and accurately as possible. The stimuli were presented using the E-Prime 2.0 software. Each trial began with a fixation point that remained on the screen for 500 ms. Afterward, a word appeared in the same position, remaining on the screen until the child responded. Vocal reaction times (vRTs) were recorded using a voice key (S-R-Box), which tracked the onset of the vocal response. The experimenter manually noted pronunciation errors, and all responses were tape-recorded for offline verification.

The words and pseudo-words were presented in a mixed order, randomized for every child. To reduce fatigue, the 60 stimuli were divided into two blocks of 30 items, with a brief pause between the blocks. Nine practice stimuli were presented before the experiment. To prevent priming pseudo-words from the words they were derived from, words and derived pseudo-words did not appear in the same block.

### 4.2. Results

Invalid trials and outliers were 2.49% for children with dyslexia and 1.27% for typically developing children.

Only vRTs corresponding to correct responses were analyzed. Self-corrections and waivers were considered errors, and the corresponding vRTs were not included in the analyses. Invalid trials and outliers were 2.43% for control readers and 8.14% for children with dyslexia.

#### 4.2.1. Test of Global Components

**Figure 3:**
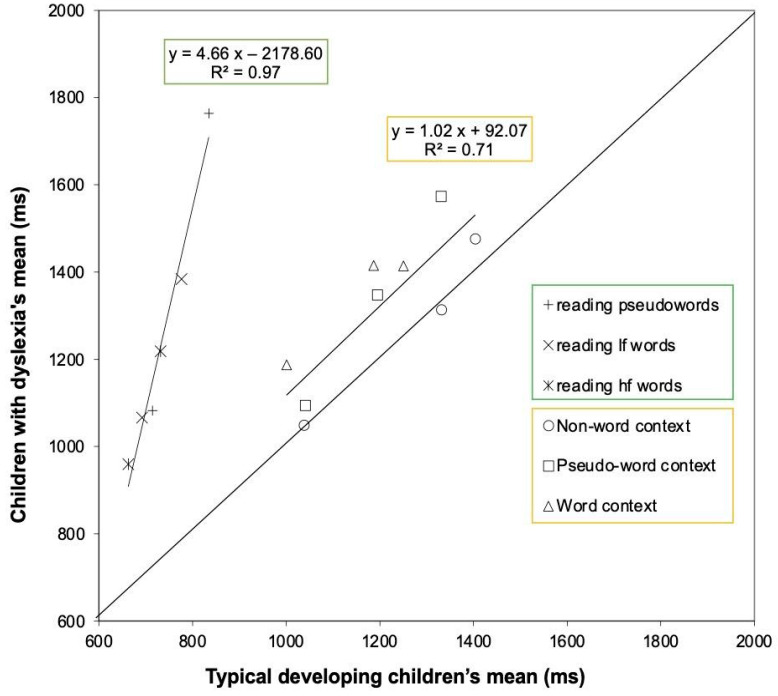
The means of the children with dyslexia are plotted as a function of those of typically developing children (Brinley plot). Separate regression lines fit the data for the reading conditions (Experiment 1B) and for the data from the Reicher–Wheeler paradigm (Experiment 1A). The dashed diagonal line (slope 1) represents equal RTs for children with dyslexia and controls.

Before analyzing specific effects, we examined the data for the potential presence of global components in the differences between the two groups of children. The RAM [12] predicts various linear relationships to define the existence of global components in the data. We initially tested the prediction of a linear relationship between the means of the two groups for conditions that varied in overall information-processing rate. The condition means for dyslexic and skilled readers are plotted against each other in Figure 3, separately for each experimental condition, in the reading task (Experiment 1B) and the Reicher–Wheeler paradigm (Experiment 1A).

Most data points are above the diagonal line, which indicates the benchmark for the identical performance between the two groups; thus, children with dyslexia tended to be slower than typically developing readers across all conditions. A two-factor solution was chosen: one regression line fitted all the reading conditions (y = 4.66x + 2178.60), and another fitted all the letter recognition conditions from the Reicher–Wheeler paradigm (y = 1.02x + 92.07). For the reading conditions, the linear relationship between the means of the two groups explained a large portion of the variance (97%). The slope of the regression was notably high (b = 4.66), indicating that the difference between the two groups steeply increased as task difficulty increased (over-additivity effect). This analysis suggests the presence of a global factor in the reading data, highlighting a severe deficit in children with dyslexia compared to typically developing readers for this task. In contrast, in the Reicher–Wheeler paradigm, the percentage of variance accounted for by the regression line was moderate (71%), and the slope was close to one (b = 1.02), indicating no over-additivity effect in this instance. The small difference observed between groups is reflected in the intercept (i.e., a constant value), which was approximately 90 ms.

**Figure 4:**
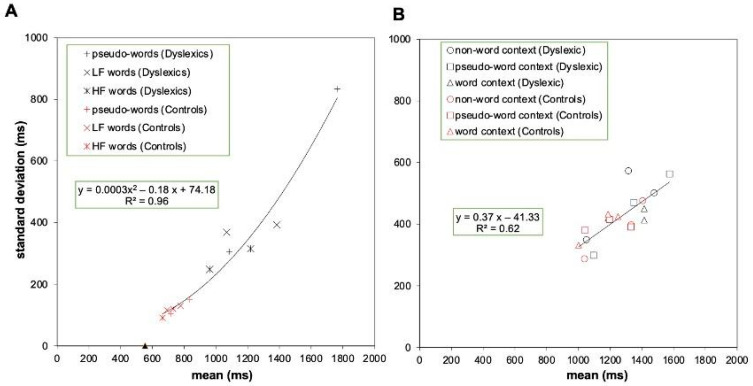
Standard deviations for each group and condition are reported as a function of the corresponding means (data from Experiment 1). Different symbols refer to data of different groups and conditions: (**A**) Data for the reading conditions: a second-order polynomial solution is reported. The black triangle marks the intercept on the x-axis of the linear solution. (**B**) Data from the Reicher–Wheeler paradigm: a linear regression is reported.

Successively, we tested the prediction of a linear relationship between the means and standard deviations in the same conditions. Based on the DEM [13], the data of the two groups are plotted separately; however, a single fit is expected to account for all experimental data. Again, a two-factor solution accounts best for the data. Figure 4A reports the data of the two groups for the reading tasks, and Figure 4B for the data of the Reicher–Wheeler paradigm.

In the case of the reading task, a linear fit accounts well for the data (y = 0.60 x − 334.63; R^2^ = 0.92). However, as predicted by DEM (Equation (7); [13], p. 271), a second-order polynomial accounts best for the data (y = 0.0003x^2^ − 0.18x + 74.18; R^2^ = 0.96), and this fit is presented in Figure 4A. The quadratic component expresses the tendency of SDs to flatten for conditions with the shortest RTs (either because of easy conditions, very fast participants, or both). According to the DEM [13], the intercept on the *x*-axis marks the sensory–motor compartment of response. Based on the linear regression fit, this was 552 ms.

A different linear regression line accounts for the data of the Reicher–Wheeler paradigm (Figure 4B). The slope of the regression was less steep (0.37), and the percentage of variance explained was lower for the conditions of the Reicher–Wheeler paradigm (62%). This percentage did not change when applying a second-order polynomial solution. The intercept on the *x*-axis was 111 ms.

When the influence of a global factor is present in the data, Faust et al. [12] recommend analyzing specific effects by comparing parametric analyses, such as ANOVAs, on both raw and z-transformed data. In this study, we focused on examining the presence of global components in the reading and the Reicher–Wheeler paradigm data. Since a global factor was identified in the reading data, we conducted the recommended analyses, which are detailed in Appendix A. In contrast, the Reicher–Wheeler paradigm data did not show evidence of over-additivity; so, these results were not analyzed further.

#### 4.2.2. Comments

The results showed a PSE in the case of accuracy data of similar size in the two groups of children. Thus, accuracy in letter discrimination in young Italian readers, and remarkably also in children with dyslexia, was influenced by the ortho-tactic regularity and/or by the pronounceability of the letter string. This finding is consistent with Marinelli et al. [15], indicating no substantial effect of list composition; i.e., the PSE was similar whether stimuli were mixed with lexical stimuli or not (Hypothesis 1). Therefore, the PSE does not appear to depend on expectation and/or associated lexical activation. The results were less clear in the case of RTs, where the PSE was not significant, inconsistent with Marinelli et al. [15], who found a significant PSE only in the case of typically developing children. This pattern is generally consistent with the literature, where accuracy is used more often than time measures to detect the PSE. Moreover, group differences in RTs were smaller in the present study using a Reicher–Wheeler paradigm with blocked conditions than in the study with mixed conditions by Marinelli et al. [15].

There was much less evidence for the WSE, taken as the difference in letter recognition between the word and pseudo-word contexts, compared to the PSE. The WSE did not reach a significance level in RTs and accuracy for both groups. This finding is inconsistent with Marinelli et al. [15], who found a WSE only in children with dyslexia for accuracy (while for latencies, the effect was never present).

The strong position effect found in children with dyslexia indicated their tendency to process non-words and pseudo-words letter by letter (Hypothesis 5) and is in line with data on the parceled scanning observed in these children [5]. However, with stimuli with a low degree of familiarity (i.e., non-words), also typically developing children showed the same position effect as children with dyslexia, with difficulty in detecting right-end characters. Both groups did not show a position effect in detecting letters in the word context, index of parallel processing, and an advantage when the top-down facilitation of the lexical representation is available. On the other hand, several studies on Italian children with dyslexia have shown that despite a deficit in lexical expansion, they were facilitated in processing stimuli when a greater lexical activation was available [11].

A general question of the study was whether performance in the Reicher–Wheeler task would generate global group differences as reported for reading tasks (Hypothesis 2). The reading tasks used in Experiment 1B yielded global differences in performance as previously reported with similar materials (e.g., [11,14]): in keeping with the predictions of the DEM [13], children with dyslexia were severely impaired compared to typically developing readers and the group differences were greater for more difficult conditions, over and above the specific effects due to frequency and lexicality (over-additivity effect). Furthermore, the interindividual variability (SD) grew considerably as a function of condition difficulty, over and above the effect of specific stimulus conditions. In a re-analysis of several experimental studies on vRTs to reading aloud, we noted that the parameters of the regression linking SDs with condition means were considerably higher than those reported by Myerson et al. [13] for a variety of other timed tasks (i.e., a slope of 0.30 and an x-intercept of 300 ms), marking the unicity of the reading task [14]. The present data align well with these parameters. The slope of this linear regression was 0.60 (0.66 in the re-analysis by Zoccolotti et al. [14]), and the intercept on the *x*-axis was 552 ms, a value in line with that reported in our previous re-analysis (482 ms).

In the case of the Reicher–Wheeler paradigm, group differences were generally much smaller (and not significant) than those observed in the case of reading tasks. When the DEM was applied to the time measures, group differences in RTs did not grow as a function of condition difficulty, as expected in the case of a global factor and an over-additivity effect. Indeed, the slope of the linear regression was near unity. Furthermore, the relationship between condition means and standard deviations was different from the reading tasks and closer to the parameters reported by Myerson et al. [13].

In the case of a closed scale, such as accuracy, the DEM cannot be applied, but some information is derived from an analysis of the effect sizes in the various ANOVAs. Thus, the effect sizes of the group factor in the Reicher–Wheeler paradigm were approximately one-fourth of those for the reading tasks (i.e., η^2^ = 0.09 for Experiment 1A; η^2^ = 0.42, for the reading tasks in Experiment 1B).

## 5. Experiment 2

In the classical Reicher–Wheeler paradigm used in Experiment 1A, the ability to maintain the multi-element string in the working memory buffer [64,65] is crucial until a response is made. The memory trace for this item can be strengthened through multiple forms of encoding. Thus, if an item can be encoded using both phonological and visual codes, it is easier to remember than an item that cannot be phonologically translated. The results observed with non-words generally support this perspective.

A search paradigm can be a helpful tool for distinguishing the impact of lexical activation on letter detection from the potential influence of memory processes. In this case, the target letter is presented before the multi-element string, and participants must determine whether the target letter appears in the string. This method requires participants to retain only one element (the target letter, not the entire string) in memory while scanning the orthographic string for that letter. It is important to note that this task differs from the previous experiment as it directly assesses children’s accuracy and RTs when dealing with orthographic strings of varying familiarity rather than through the indirect effects of contextual influences on letter processing. We expect that the activation of whole-form representations will inhibit the detection of competing letters due to backward activation of the letter nodes associated with the word, regardless of the letter’s position. However, for letter strings likely processed through using small grain-size units (such as pseudo-words and, especially, non-words), identifying a letter may be easier and more influenced by the letter’s position. Experiment 2 was conducted to verify these hypotheses.

### 5.1. Materials and Methods

#### 5.1.1. Participants

Twelve children with dyslexia and 13 typically developing children who had already taken part in Experiments 1A and 1B also participated in Experiment 2.

#### 5.1.2. Stimuli

Words, pseudo-words, and non-words were those of Experiment 1A.

#### 5.1.3. Procedure

**Figure 5:**
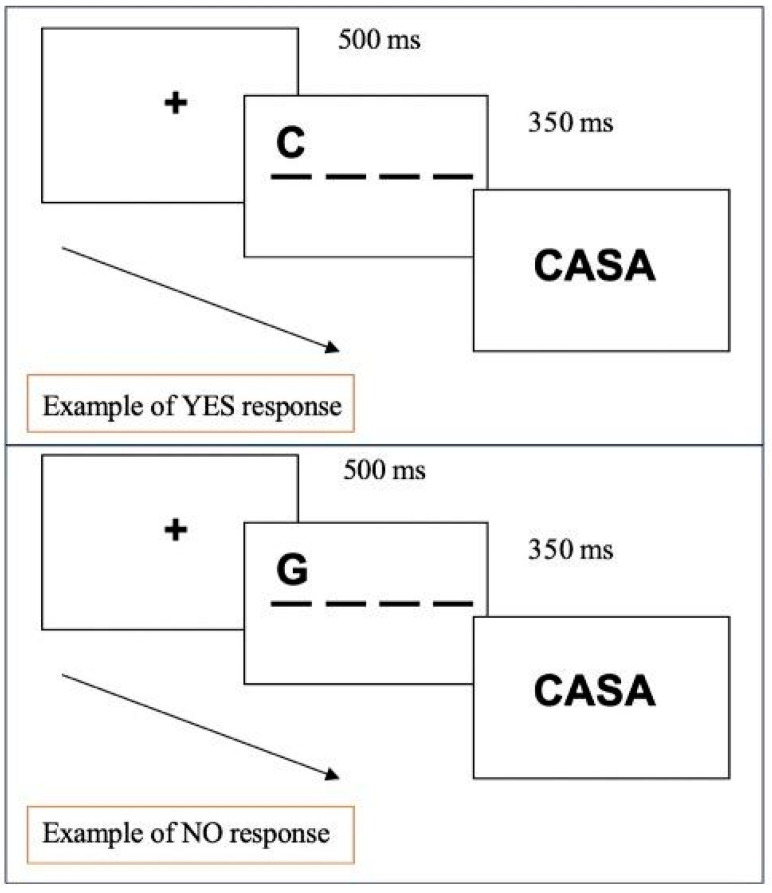
Experiment 2: Time-course of the trial: examples of YES and NO responses.

The trial sequence started with a get-ready display (500 ms), followed by the presentation of the target-letter display in the correct position for 350 ms and by the multi-letter array (word, pseudo-word, or non-word), which lasted until the child produced his/her response by pressing one of two buttons: YES if the target letter was in the string, NO if it was not. Figure 5 represents an example of the trial sequence in the case of YES and NO responses.

### 5.2. Results

Outliers and invalid responses were few: 0.19% for typically developing readers and 0.78% for children with dyslexia.

#### 5.2.1. Accuracy

**Figure 6:**
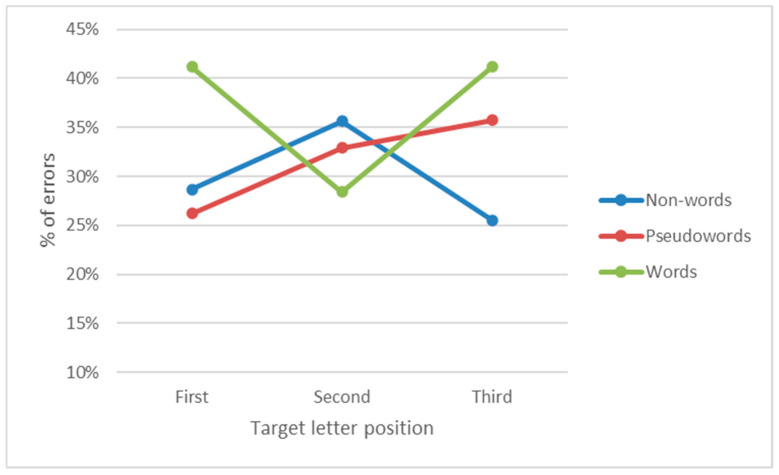
The type of display by position interaction on percentages of errors (Experiment 2).

The ANOVA on errors showed the main effect of the type of array (F_2,46_ = 18.47, *p* < 0.001, η^2^ = 0.45): more errors were present while searching the target letter in the word array (36.9%) than in the pseudo-word (31.5%; *p* < 0.001; SMD = 0.42) and non-word (29.7%; *p* < 0.001; SMD = 0.49) arrays, which did not differ from each other. The main effect of the group factor was not significant (F_1,23_ = 0.00, n.s.; both groups made 32.7% of errors), as well as all interactions of the group effect with the other factors. The type of display by position interaction (F_4,92_ = 14.42, *p* < 0.001) was significant (Figure 6), indicating a different positional trend for the three types of arrays. Letter recognition errors in the first position were reliably higher while searching for the target in a word than in a pseudo-word (*p* < 0.01; SMD = 1.08) or a non-word (*p* < 0.01; SMD = 0.99). In the third position, letter recognition errors in the non-word context were fewer than in the word (*p* < 0.01; SMD = −0.96) and pseudo-word (*p* < 0.01; SMD = −0.61) contexts. In the second position, differences among conditions were generally smaller; however, the difference between the word and non-word arrays was significant (*p* < 0.05; SMD = −0.37).

#### 5.2.2. Reaction Times

The group effect was not significant (F < 1, n.s.): groups had similar RTs (1460 vs. 1484 ms for typically developing and dyslexic children, respectively). RTs did not vary appreciably as a function of the type of array (F < 1; 1458, 1478, and 1481 ms for non-word, pseudo-word, and word arrays, respectively) as well as a function of position (F < 1; 1476, 1447, and 1494 ms in the first, second, and third position, respectively).

#### 5.2.3. Test of Global Components

There was little evidence of global influences on the data of Experiment 2. The Brinley plot indicated no relationship between the condition means of the two groups (y = −0.029x + 1526; R^2^ = 0.0004). Furthermore, there was no relationship between the condition means and the corresponding SDs (y = −0.008x + 114.4; R^2^ = 0.005). The small range across experimental means may have contributed to this outcome (for children with dyslexia, Min = 1359 ms and Max = 1550, range = 211; for controls, Min = 1382 ms and Max = 1590, range = 207)

#### 5.2.4. Comments

In a newly devised search paradigm, we found that the expected interference of lexical representation affected accuracy: recognizing a target letter was more challenging when it appeared within a word compared to a pseudo-word or a non-word. This effect was observed similarly in both groups of children (Hypothesis 3). Therefore, at least for short, high-frequency words, children with dyslexia automatically activate lexical entries, even when not explicitly required by the search task.

There was no overall difference between searching for a letter within a pseudo-word and a non-word. However, the three array conditions revealed a distinct pattern of positional effects. In the case of non-words, the absence of vowels may play a selective role. The pre-activating vowels in the second position can be beneficial for words and pseudo-words, while for non-words, it becomes necessary to inhibit the vowel, making the search task more difficult. This might explain why the second position in words was associated with the lowest error rate—there are fewer vowels than consonants, which reduces memory load. For pseudo-words, the backward inhibition from word representations sharing the first letter with the stimulus may account for the high error rates in the second and third positions.

Analyzing reaction times (RTs) did not reveal any significant main effects or interactions, suggesting that latencies were not a sensitive measure for capturing group, context, or positional effects in the letter search task. Additionally, there was no evidence of global components within the data, and the limited range of conditions may have contributed to this outcome.

Overall, the search task proved more effective than the Reicher–Wheeler paradigm in demonstrating lexical influences on letter recognition. However, it was less effective in highlighting the role of ortho-tactic regularity, which was influenced by positional effects. Both children with dyslexia and typically developing readers were less accurate when searching for a letter within a word than when searching within non-lexical arrays of letters. Therefore, the preserved lexical activation in children with dyslexia is the key finding of this experiment.

## 6. Experiment 3A

In this experiment, we used symbol strings to assess the putative specificity of orthographic effects compared with non-orthographic materials. In Experiment 3A, the Reicher–Wheeler paradigm was adopted, and in Experiment 3B, the search paradigm (as in Experiment 2).

### 6.1. Materials and Methods

#### 6.1.1. Participants

The same 17 children with dyslexia and 24 typically developing readers, who took part in Experiment 1, participated in this experiment.

#### 6.1.2. Stimuli

Strings of Greek letters were used, selecting only letters that do not resemble Italian letters (such as ε or ɳ) and are not commonly used as mathematical symbols (such as π). Like Experiment 1A, the strings were composed of four letters each. A total of sixteen items were generated for each position. Only the targets referring to the first, second, and third positions (N = 48) were considered experimental items, while the strings with targets in the fourth position were considered fillers.

#### 6.1.3. Procedure

The same general procedure used in Experiment 1A was followed. Ten training stimuli were presented at the beginning of the experimental session. The stimuli were presented in a single block in a completely randomized order.

### 6.2. Results

Outliers and technical failures, which were excluded from the analysis, accounted for 2.35% of the cases in children with dyslexia and 1.74% in typically developing children. The ANOVA on errors showed no significant effect of the group factor (F_1,39_ = 0.15, n.s., η^2^ = 0.00). The main effect of position approached significance (F_2,78_ = 3.05, *p* = 0.053, η^2^ = 0.07): errors in the third position were higher (46.1%) compared to the first (39.9%, *p* < 0.05; SMD = 0.42) and second (40.0%, *p* < 0.05; SMD = 0.52) positions. The main effect of the group (mean errors = 41.4% vs. 42.6% for typically developing and children with dyslexia, respectively) and the interaction with position were not significant.

The ANOVA on RTs did not show the effect of the group (F_1,39_ = 0.01, n.s.) nor were there any other significant main effect or interaction. RTs were similar between typically developing readers (1448 ms) and children with dyslexia (1468 ms), as well as across the first (1448 ms), second (1418 ms), and third (1541 ms) positions.

## 7. Experiment 3B

### 7.1. Materials and Methods

#### 7.1.1. Participants

The twenty-five children taking part in Experiment 2 also participated in Experiment 3B.

#### 7.1.2. Stimuli

Same as in Experiment 3A.

#### 7.1.3. Procedure

The search paradigm was used, following the same procedure as Experiment 2.

### 7.2. Results

Outliers and invalid trials were 2.78% for children with dyslexia and 2.56% for typically developing children.

In the ANOVAs, the group effect was negligible for both error (F_1,23_ = 0.51, n.s., η^2^ = 0.02) and RT data (F_1,23_ = 0.06, n.s., η^2^ = 0.00). Typically developing readers made 25.3% of errors with a mean RT of 1324 ms, while children with dyslexia made 18.7% of errors with a mean RT of 1357 ms. The position effect was also not significant for both error (F_2,44_ = 0.98, n.s., η^2^ = 0.04) and RT data (F_2,44_ = 0.58, n.s., η^2^ = 0.03). The error rates for the positions were as follows: first position: 24.2% of errors with an RT of 1344 ms; second position, 21.6% of errors with an RT of 1295 ms; and third position, 20.1% of errors with an RT of 1383 ms.

To test for global components, the results of Experiments 3A and B were collapsed. Even in this way, there was very little evidence for the presence of global components in the data. The Brinley plot, contrasting the performance of children with dyslexia and controls across conditions, revealed sparse data with a slope of less than one and very little variance accounted for (y = 0.45x + 781.12; R^2^ = 0.17). The regression contrasting means and SDs yielded a very low slope and again a low explained variance (y = 0.12x + 44.80; R^2^ = 0.31)

### 7.3. Comments

Children with dyslexia did not show a deficit in detecting a symbol compared to typically developing children, regardless of whether the symbol string preceded or followed the target symbol (Hypothesis 4). However, both groups found the task more challenging (longer RTs and higher percentage of errors) when the symbol string preceded the target symbol (as seen in the classical Reicher–Wheeler paradigm), which resulted in longer response times and higher percentages of errors. This difficulty is likely due to the need to hold a string of unfamiliar symbols in memory in the former case; however, this asymmetry did not affect the presence or magnitude of the differences between the groups.

These findings align with other literature indicating that children with dyslexia exhibit impairments in tasks involving letters but not symbols [34]. Furthermore, Shovman and Ahissar [35] reported that adults with dyslexia were unimpaired in processing unfamiliar letter-like symbols under various conditions, such as different letter sizes, crowding, and white noise. In contrast, Pammer et al. [20] found that children with dyslexia struggled when asked to process a string of non-alphabetic but letter-like symbols. The number of elements processed simultaneously seems to be a key factor, as previously discussed in relation to linguistic stimuli in Experiment 1A.

Overall, Experiment 3 indicated that the performance of children with dyslexia in processing symbols (whether in a Reicher–Wheeler-like or search paradigm) could not be distinguished from that of typically developing readers. This finding highlights the selective nature of the processing delays experienced by children with dyslexia.

## 8. Discussion

While children with dyslexia are impaired whenever they must process a string of letters, their performance is surprisingly spared when processing single letters. This dissociation was first reported by Katz and Wicklund [17,18] and has since been supported by sensitive letter recognition tests [19]. In this study, we explored the interaction between letter and letter string processing. To this aim, we used variations of the 2AFC paradigm to investigate how letter-word and pseudo-word interactions vary as a function of reading expertise. Specifically, we employed two different paradigms: the Reicher–Wheeler procedure and a search task. These approaches allowed us to examine the influence of orthographic and lexical factors on letter recognition as well as the specificity of these effects for orthographic stimuli.

The two tasks proved differentially sensitive to evidence effects due to lexical or orthographic activation. Although originally developed to examine the role of lexical activation in the WSE, the Reicher–Wheeler procedure was more effective in showing the PSE than the WSE effect, even in a blocked condition, where we expected a weaker lexical activation for non-lexical stimuli than in the mixed condition used in Marinelli et al. [15] (Hypothesis 1). The present pattern of findings shares several similarities with the previous results on French children reported by Grainger et al. [40]. They found a strong PSE effect in both dyslexic and reading-matched control children but no WSE for either group of children (while the WSE was present with the same type of stimulus materials in a group of adult readers). They proposed that the joint presence of PSE and absence of WSE favors a sublexical–orthographic interpretation based on the greater familiarity of letter combinations in pronounceable pseudo-words compared to unpronounceable non-words. Thus, it would be the presence of units that code relative letter position in a string that is the base of the PSE. Pseudo-words provide letter clusters representing typical orthographic contexts for a given letter in a given position [40]. In the present study, non-words provide only partial information in this respect (namely, information about bigrams). By contrast, the facilitating role of lexical activation producing the WSE would emerge only at more advanced stages of the reading experience.

Grainger et al. [40] also noted that the WSE effect was present in adult readers and proposed that the effect is present only after considerable reading experience. As we did not test adults, we do not have direct evidence on this point. Another option, based on the results of our experiments, is that the WSE is hard to detect because of a range limitation. Indeed, the proportion of errors in the case of a pseudo-word context was relatively small, and there was little room for additional improvement. Some indication in this sense derives from data from Marinelli et al. [15]. The WSE effect was significant only in the case of the lowest performance, i.e., in children with dyslexia in the case of mixed lists [15]. Note that the two interpretations proposed may both be at work; namely, a range restriction may make it difficult to obtain an effect, which consolidates only after several years of experience with the lexicon, as suggested by Grainger et al. [40].

By contrast, the search task used in Experiment 2 was particularly effective in detecting lexical influences and, less so, those due to orthographic regularity. Both groups of children were delayed when searching for a letter within a word rather than a pseudo-word or non-word (Hypothesis 3). To the best of our knowledge, search tasks have always been used with strings of random letters. Contrasting lexical and non-lexical arrays seems to be an effective way to detect lexical effects in children. The influence of orthographic regularity was somewhat less clear. Indeed, it is expressed most clearly in relationship to the impact of the target position. Consistent with the literature, the accuracy measures were generally more effective in detecting the experimental effects (and the group differences).

### 8.1. Testing for Global Components

One of the goals of the study was to determine whether performance in the Reicher–Wheeler and search tasks would reveal global group differences similar to those previously reported for reading tasks.

The data from the reading task (Experiment 1B) largely confirmed earlier findings, indicating that children with dyslexia exhibited significantly impaired performance (Hypothesis 2). This impairment was strongly correlated with the level of difficulty presented in the experimental conditions beyond the specific characteristics of those conditions. Indeed, there was minimal evidence of specific residual effects once the influence of global components was adjusted using z-transformed data (see Appendix A), as suggested by the RAM [12]. Furthermore, consistent with the predictions of DEM, the interindividual variability (SDs) increased sharply as a function of task difficulty, as is typical of reading-aloud tasks [13].

In contrast, there was little evidence of global components affecting performance on the Reicher–Wheeler task. The Brinley plot comparing the performance of the two groups revealed a slope very close to unity, indicating that the group differences were independent of the overall level of difficulty of the tasks. Moreover, the interindividual variability in this task only increased moderately as a function of condition difficulty, further highlighting its distinction from the pattern observed in reading-aloud tasks. Additionally, there were few indications of global components in the search tasks, whether for letters (Experiment 2) or symbols (Experiment 3).

These data are generally in keeping with previous observations by De Luca et al. [16], indicating that children with dyslexia were only mildly affected in tasks requiring the naming or matching of individual letters, bigrams, or two-letter syllables, and no over-additivity effect was present for these tasks (see also [15], for similar data). Therefore, it appears that the global factor accounting for the impairment of children with dyslexia is present when the child processes a string of letters (whether a word or a pseudo-word) in parallel, not when the task concerns one or two letters.

The present results add to this picture that, even though the processing of a letter string is delayed in these children, they can take advantage of the ortho-tactic information deriving from such processing to tune the analysis of a subsequent isolated target letter. This differentiation can be appreciated most clearly by comparing the performance in reading four-letter pseudo-words with that in recognizing a target letter in the presence of a four-letter pseudo-word context in Experiment 1A. In the first condition, children with dyslexia were severely impaired in both accuracy and speed; in the second, they were more accurate than in the case of a four-letter non-word context (i.e., they had a PSE), and the group difference with typically developing children was quantitatively quite small. Therefore, only the online processing of a letter string is impaired in these children, not the ability to use the ortho-tactic information derived from the letter string.

### 8.2. Letter Position Effects

In the non-word context, skilled fourth graders show the typical first-position effect found in adult skilled readers: the first letter is processed with a high level of accuracy, whereas the second and third letters are both associated with lower accuracy and do not differ from each other [31,66]. In this context, children with dyslexia show a first-letter advantage like young and adult skilled readers but also show an appreciable impairment when the 2-AFC must be carried out on the third letter of the string compared to the second one (third-position disadvantage effect) (Hypothesis 5). This specificity of children with dyslexia also appears, even though attenuated, in processing pseudo-word strings and even more in the word context. Again, responses on the third position are less accurate than responses on the first two letters of the string, which do not differ. In the pseudo-word context, typically developing children show a first-letter advantage, while no letter-position effect is found in the word context. The homogeneity of accuracy associated with the letter positions in a word context is observed in both groups of children, even though children with dyslexia show a very tiny third-position disadvantage effect.

The analysis of RTs provides interesting insights into how letter strings are processed: There are no differences between skilled readers and children with dyslexia and, in every context examined, a consistent first-letter position effect is observed. A linear increase in time from the first to the third position may only be seen in the non-word and pseudo-word contexts, affecting both groups similarly.

Data from Experiment 3 show that when children must process perceptual strings of symbols, their accuracy drops dramatically, approaching chance levels (Hypothesis 4). Position effects disappear both in accuracy and latency, and these effects do not interact with the group factor. The symbol string context can be regarded as a baseline for the visual perceptual system. The differences observed in this condition compared to the performance measured in Experiment 1A might reflect the impact of learning to read on processing strings of elements. Based on these results, the first-position advantage and third-position disadvantage observed in the accuracy analysis, along with the first-position advantage and linear increase in latency for non-words and pseudo-words in the RT analysis, may be consequences of the reading acquisition process.

Data on accuracy in non-word and pseudo-word contexts support Tydgat and Grainger’s [31] assertion that learning to read helps refine the receptive fields for letters, thereby reducing crowding effects, especially for characters on the right of fixation. Typically developing children do not exhibit any differences between letters in the second and third positions; instead, they show a clear advantage for letters in the first position. It can be hypothesized that their receptive fields are small enough to focus on the target letter and just one character on the left. According to this hypothesis, the advantage of the first position arises from the absence of interference from the left side. In contrast, the similar levels of accuracy observed for letters in the second and third positions can be attributed to the interference caused by a single letter on the left.

The disadvantage seen in the third position for children with dyslexia suggests a different perspective. It may be inferred that their receptive fields are too large, making them susceptible to disruptive crowding effects. Thus, the number of flanking characters on the left increases, and the accuracy of letter identification decreases. Consequently, identifying the letter in the third position becomes more challenging because it is influenced by two preceding letters, whereas the letter in the second position is influenced by only one letter. The higher number of fixations observed in children with dyslexia during reading tasks [5] may stem from their difficulty in recognizing a single letter within multi-element strings, likely due to their atypical receptive fields.

The absence of letter position effects in a word context for typically developing children suggests that they may benefit from lexical activation. Thus, backward activation from lexical entries triggers a whole-word perception, potentially reducing crowding effects. In contrast, children with dyslexia show only a slight third-position disadvantage effect within a word context.

Analysis of RTs reveals a strong first-position effect for all children in every context. Both typically developing children and children with dyslexia demonstrated a tendency to begin processing letter strings from the initial letter, which (in left-to-right languages) is the most informative about the lexical identity of the target word. However, when limited to non-words and pseudo-words, a left-to-right scanning pattern can be observed, as modelled by Coltheart et al. [9]. It may be hypothesized that this sequential mechanism is effective in mapping orthography to phonology and is also employed when dealing with unpronounceable strings of consonants (such as CCCC). Conversely, Marinelli et al. [53] found that Italian children tended to memorize orthographic stimuli using a phonological strategy, contrary to English children, who relied more on a visual strategy. Furthermore, it is relevant to note that the present study was conducted on a highly consistent orthography. In such cases, a partial activation of whole-word representations could support accurate lexical retrieval, even among children with reading impairment. Therefore, it would be informative to investigate these patterns in children speaking an inconsistent orthography, such as English. Regarding word strings, the Dual-Route Cascade framework [9] posits that direct activation of lexical representations overtakes the grapheme-to-phoneme conversion process, eliminating differences among all letter positions except for the first.

## 9. Conclusions

For all letter recognition tasks, there are either small or no group effects, as indicated by small η^2^ and by the application of the RAM and DEM. Moreover, the group effect was not significant for search tasks or symbol processing. Letter tasks prove generally quite challenging for children, resulting in relatively slow RTs. However, this increased difficulty does not lead to a substantial group effect but rather a minor one.

Overall, children with dyslexia exhibited the following: (i) preserved lexical activation in the search task; (ii) maintained sensitivity on ortho-tactic regularity; (iii) minimal or absent group differences in letter discrimination tasks and no group differences in symbol processing; (iv) severe difficulties in reading both words and pseudo-words; and (v) a slight deficit in the classical condition of the Reicher–Wheeler paradigm, but not in the search task. This latter finding highlights that the deficit is not due to an inability to scan the letter string, as children can correctly identify a single letter within a string. Compared to the classical Reicher–Wheeler paradigm, the search task reduces memory loading and time pressure since it requires online processing of the letter string.

Children with dyslexia are severely impaired when processing multiple arrays of information, such as those encountered in reading. In contrast, their performance is unaffected or only slightly impacted when dealing with single targets. When processing such stimuli, their performance is either unaffected or minimally influenced. In this vein, searching for conditions that are or are not significant (as carried out in 2AFC literature) may not be the most effective way to identify the specific deficits associated with dyslexia. The processing of a letter string is slowed (and inaccurate) but somehow proceeds in the information processing stream. Thus, it allows for activating ortho-tactic and lexical information, which can facilitate or hinder letter processing, even in dyslexic children, depending on the stimulus conditions. Further studies will be necessary to replicate these findings in a larger sample of dyslexic children and control for individual differences in socioeconomic level or educational factors, as well as cognitive effort or fatigue (due to the high number of experimental tasks tested on the same sample).

## Figures and Tables

**Table 1:** Performance of children with dyslexia and typically developing readers on the Raven CPM Matrices, and on phonological and visual attention span tests ^a^.

	Typically Developing Readers		Children with Dyslexia				
	Mean	SD	Mean	SD	% of Path. Perform	Student t/Mann–Whitney U	*p*
Reading speed	−0.10	0.57	−1.75	1.25	59%	t = 5.73	<0.0001
Reading errors	−0.03	0.79	−2.99	1.19	100%	U = 141	<0.001
Reading comprehension	0.04	0.26	−0.27	0.43	23%	t = 2.84	<0.01
Raven CPM test	0.04	0.88	0.03	0.71	--	t = 0.04	n.s.
Blending test: Words	0.73	0.94	0.05	1.31	5%	U = 326	0.077
Blending test: pseudo-words	0.64	1.10	−0.53	1.21	16%	t = 3.21	<0.01
Repetition of Pseudo-word Series	0.87	1.15	0.17	0.85	5%	t = 2.12	<0.05
Phonological Span	0.61	1.21	0.06	0.66	--	t = 1.65	n.s.
Visual Attentional Span	0.44	0.70	−0.14	0.43	--	t = 3.01	<0.01

^a^ Values indicate z scores compared to normative values (negative values indicate lower performance). SD = Standard Deviation; % path. perform: percentage of children who score less than 1.65 standard deviations below the mean according to normative values. *t*-tests were performed for normally distributed variables, while Mann–Whitney U tests were for data deviant from normality according to the Kolmogorov–Smirnov test.

## Data Availability

The raw data supporting the conclusions of this article will be made available by the authors on request.

## Appendix A

### Appendix A.1. Test of Specific Effects

In the presence of a global factor in the data, Faust et al. [12] suggest comparing parametric analyses (such as ANOVAs) on raw vs. z-transformed data. Interactions that are significant in both the raw score and z-transformed score analyses indicate the selective influence of a given parameter; by contrast, interactions that are significant only with the raw data but not with the z-transformed values indicate the presence of a spurious interaction (i.e., due to the presence of an over-additivity effect). Therefore, raw data for the reading task were transformed into z scores by taking each individual’s condition means, subtracting their overall mean, and dividing it by the standard deviation of their condition means. This transformation (possible for time but not accuracy measures) re-scales individual performance to a common reference; hence, it allows controlling for global components while it preserves the information regarding individual variability across experimental conditions.

As a global factor was not detected for the conditions of the Reicher–Wheeler paradigm, the z-score transformation was not used beyond the standard RT analyses described above (see Experiment 1).

As for the reading task, mixed ANOVAs were carried out on the percentages of errors, as well as mean vRTs and z-transformed values; these two latter analyses will be presented together to appreciate the change in results in the presence of over-additivity. In all analyses, the group (children with dyslexia vs. typically developing children) was the between-participant factor, and type of stimulus (high-frequency, low-frequency words, and pseudo-words), and length (4-, 6-letter stimuli) were the within-participant factors. Significant interactions were explored with the a posteriori Duncan test.

#### Appendix A.1.1. Accuracy

The ANOVA on error data showed the main effects of group (F_1,39_ = 28.59, *p* < 0.0001, η^2^ = 0.42), length (F_1,39_ = 64.43, *p* < 0.0001, η^2^ = 0.62), and type of stimulus (F_2,78_ = 46.70, *p* < 0.0001, η^2^ = 0.54), as children with dyslexia made more errors (19.0%) than typically developing children (6.3%), pseudo-words were read with more errors (20.3%) than low-frequency (11.8%) and high-frequency (5.9%) words, and the percentage of errors increased from 4-letter (6.7%) to 6-letter (18.6%) stimuli.

Length interacted with type of stimulus (F_2,78_ = 10.71, *p* < 0.0001): larger length effects were present for pseudo-words (difference between 6- and 4-letter stimuli = 19.4%) than for low-frequency (difference = 10.2%) and high-frequency (difference = 6.3%) words. The group by length interaction (F_2,39_ = 8.33, *p* < 0.01) indicated a larger length effect in children with dyslexia (difference between errors for 6- and 4-letter stimuli = 16.2%) than in typically developing children (difference = 7.6%). The group by type of stimulus interaction (F_2,78_ = 12.34, *p* < 0.0001) indicated a larger group difference in reading pseudo-words (difference = 21.36%) than low-frequency (difference = 7.81%) and high-frequency (difference = 9.21%) words.

#### Appendix A.1.2. Reaction Times

The ANOVA on vRTs showed the significance of the main effects of the group (F_r1,39_ = 42.32, *p* < 0.0001, η^2^ = 0.52), length (F_r1,39_ = 92.84, *p* < 0.0001, η^2^ = 0.70; F_z1,39_ = 145.60, *p* < 0.0001, η^2^ = 0.79), and type of stimulus (F_r2,78_ = 23.74, η^2^ = 0.38, *p* < 0.0001; F_z2,78_ = 30.54, *p* < 0.0001, η^2^ = 0.44) factors, with longer vRTs for children with dyslexia (1246 ms) compared to typically developing children (735 ms), for 6-letter stimuli (1118 ms) compared to 4-letter stimuli (863 ms), and for pseudo-words (1099 ms) compared to low-frequency (980 ms) and high-frequency (893 ms) words.

Length interacted with type of stimulus (F_r2,78_ = 8.36, *p* < 0.001; F_z2,78_ = 4.14, *p* < 0.05), indicating a greater length effect with pseudo-words (difference between 6- and 4-letter stimuli = 401 ms) than low-frequency (difference = 201 ms) and high-frequency (difference = 164 ms) words. The group interacted with length (F_r1,39_ = 38.38, *p* < 0.0001; F_z1,39_ = 1.83, ns) and type of stimulus (F_r2,78_ = 9.32, *p* < 0.001; F_z2,78_ = 0.64, ns) in the raw data but not in z-transformed data analysis. Also, the group by length by type of stimulus interaction was significant in the raw but not in z-transformed data (F_r2,78_ = 5.29, *p* < 0.01; Fr_2,78_ = 0.50, ns). Exploring this interaction in terms of raw data indicated that group differences grew bigger for more difficult conditions but that these differences could be entirely ascribed to over-additivity.
